# Identifying protective factors for gender diverse adolescents’ mental health

**DOI:** 10.1038/s44184-023-00029-8

**Published:** 2023-07-04

**Authors:** Melissa K. Holt, Katharine B. Parodi, Frank J. Elgar, Abra Vigna, L. B. Moore, Brian Koenig

**Affiliations:** 1 Boston University Wheelock College of Education and Human Development, Two Silber Way, Boston, MA 02215 USA; 2 McGill University, 1130 Pine Avenue West, Montreal, QC H3A 1A3 Canada; 3 University of Wisconsin-Madison, Madison, WI 53726 USA; 4 Ampersand Healing, West Springfield, MA 01089 USA; 5 K-12 Associates, Madison, WI 53719 USA

**Keywords:** Education, Psychology

## Abstract

Few studies have disentangled differences in victimization exposures and mental health symptoms among gender diverse subgroups, nor considered the role of potential protective factors in ameliorating the impact of victimization on gender diverse youths’ mental health. Here we report findings from a secondary data analysis, in which we address this gap by analyzing cross-sectional survey data (*N* = 11,264 in the final analytic sample) from a population-based survey of youth in participating school districts in a large Midwestern U.S. county. Relative to cisgender youth with gender conforming expression, transgender youth and cisgender youth with nonconforming gender expression are more likely to experience victimization and severe mental health concerns. Additionally, school-connectedness moderates the association between bias-based harassment and depression for cisgender youth with gender nonconforming expression, and family support/monitoring buffers the association of peer victimization with suicide attempts among transgender youth. Findings highlight the need to better understand factors which may confer protection among gender diverse adolescents, so that in turn appropriate supports across key contexts can be implemented.

## Introduction

Increasingly, research on gender diverse (GD) individuals^1^ (defined by the National Institutes of Health to include, but not be limited to, transgender and gender nonconforming individuals) has expanded to consider the experiences of youth. National prevalence estimates are absent, but drawing from extrapolations of adult and state-level survey data, approximately 0.7–2.7% of adolescents identify as transgender and upwards of 27% identify as gender nonconforming^2–5^. Limited research, however, considers the unique experiences of gender diverse youth subgroups, given that subgroups are frequently combined into a monolithic gender diverse group and/or combined with sexually diverse adolescents. Further, few studies have considered the experiences and mental health of youth by their perceived level of gender conforming expression. This study addresses these gaps using data from a population-based sample of youth to assess the mental health and victimization experiences of transgender and cisgender youth with nonconforming gender expression, and to evaluate potential protective factors. We acknowledge that a range of umbrella terms are used to refer to transgender and gender nonconforming individuals and have selected “gender diverse” for consistency throughout.

Marked disparities between gender diverse and cisgender youth (i.e., youth whose gender identity and sex assigned at birth align) exist^6–11^. In a recent review, Tebbe and Budge (2022) highlighted that for GD individuals broadly, the most marked disparities between GD and cisgender people exist for depression and anxiety. With respect to youth populations specifically, one study found that 57.9% of transgender and gender nonconforming (TGNC) youth reported depressive symptoms compared to 21.3% of cisgender youth^3^. Other studies have found similar health disparities in substance use^5,12^, suicide attempts^6,12–16^, suicide ideation^6,12,16^, and nonsuicidal self-injury^15–17^ among TGNC adolescents. Further, gender diverse youth experience numerous challenges at school, including limited access to appropriate restrooms, not feeling as connected to school, and more absenteeism, than cisgender peers with gender conforming expression^18–21^. Transgender adolescents are more likely to experience victimization, including sexual assault, dating violence, and bullying^5^, as well as childhood sexual abuse^12^, and are disproportionally represented among adolescents who have experienced multiple forms of victimization^22^. Furthermore, transgender youth who experience additional forms of marginalization (e.g., racism, ableism) in addition to anti-transgender bias are disproportionately impacted by victimization compared to transgender peers who do not experience multiple forms of marginalization^23^. These disparities have been conceptualized through a minority stress framework^24^, acknowledging greater exposure to proximal and distal stressors among gender diverse individuals due to their marginalized identities in society^25,26^.

Only recently have studies considered protective factors among gender minority youth^3,25–28^. Olson and colleagues^27^ found that children who had socially transitioned—considered as a protective factor and proxy for family support—did not report the same high depression and anxiety rates that had been documented in broader transgender youth samples. This finding was supported by a state-wide survey that found parent-connectedness was associated with lower odds of self-reported depression, substance use, and suicidality among gender diverse youth compared to those reporting lower levels of parent-connectedness^28^. Recent longitudinal findings have also highlighted the salience of social support and family functioning as factors that buffer the association between lower levels of exposure to gender minority stressors and alcohol use^29^. Further, support from educational and trans communities has been identified as salient for transgender youth^30^, and greater school-connectedness has also been linked to fewer mental health concerns among GD adolescents^31^. Finally, youth whose gender was affirmed—as operationalized by chosen name use across four contexts—reported better mental health than youth who used chosen names in fewer contexts^32^. Increasingly, researchers have called for a move toward a strengths-based perspective in studies of gender diverse individuals, and to identify potential protective factors^33^.

Study aims are to: (1) examine differences in mental health and victimization prevalence between transgender and cisgender adolescents, with consideration to cisgender adolescents’ degree of conforming gender expression, and (2) examine the extent to which family support/monitoring and school-connectedness moderate associations between victimization and psychological distress, with separate models for transgender youth and cisgender youth with gender nonconforming expression. Implications of study results for researchers and practitioners are discussed.

## Methods

### Study population

Data come from the 2015 Dane County Youth Assessment (DCYA), an anonymous, web-based, cross-sectional survey of students in Wisconsin’s Dane County. In 2015, all public school districts in the county, one religious school in the county, and one additional school district partially located in the county, were invited to participate in the DCYA. The majority of school districts participated, with only two public high schools opting out of survey participation in 2015. Parents/guardians were notified about the survey and had the opportunity to review the survey at their child’s school. If they did not want their child to complete the survey, they were asked to return a signed opt-out form. Prior to completing surveys students were told the survey was optional, and students who did not assent to participate completed another activity during the survey administration time. Students completed surveys using school computers between January to March 2015, with response rates of >90%. At the majority of schools, all students were invited to complete surveys. In contrast, the large urban schools (*n* = 5) sampled approximately 50% of students, and post-stratification survey weights were applied to reflect the sociodemographic composition of these schools. Completing the survey were 13,905 high school students (unweighted distribution: *M* age = 15.9 years, SD = 1.2, 75.4% White non-Hispanic, 89% straight/heterosexual, 50.3% male, 1.4% transgender; weighted distribution: *M* age = 15.9 years, SD = 1.2, 67.3% White non-Hispanic, 88.2% straight/heterosexual, 49.3% male, 1.4% transgender) in 22 high schools and 1 juvenile detention facility. The methods were performed in accordance with relevant guidelines and regulations and approved by Boston University’s Institutional Review Board, which declared this study exempt. Reporting of this study adheres to the Strengthening the Reporting of Observational Studies in Epidemiology (STROBE) guidelines for cross-sectional studies.

### Sociodemographic characteristics questions

Students were asked “How old are you?”, with response options of 14-year old or younger, 15-year old, 16-year old, 17-year old, or 18-years old or older. Students were asked, “What is your biological sex?” Response options were male or female. A single-item asked students their race/ethnicity. Response options were collapsed to six identities for descriptive purposes: White, Black, Latino, Asian including Hmong, Multi-racial, and Another race/ethnicity. For sexual orientation, students were asked “Which of the following best describes you?” Response options were straight/heterosexual, gay or lesbian, bisexual, questioning my sexual orientation, or another sexual orientation. Age, biological sex, and race/ethnicity (collapsed to a dichotomous variable for more parsimonious models) were included as covariates in multivariable models. Sexual orientation was included for descriptive purposes and tested in initial models, but excluded as a covariate given the strong association between sexual orientation and gender nonconformity.

### Gender modality and gender expression groups

Youth were asked, “Do you identify yourself as transgender?” Response items were yes, no, and I don’t know what transgender means. Youth who responded yes were classified as transgender, and youth who responded no were classified as cisgender. Youth who responded “I don’t know what transgender means” were excluded from the analytic sample (see Supplementary Fig. 1). We use the term gender modality given that this describes the relationship between one’s gender identity and the sex they were assigned at birth (e.g., cisgender, transgender, nonbinary), in contrast to gender identity, which describes the gender with which someone identifies (e.g., girl, nonbinary, boy)^34,35^.

Respondents who did not identify as transgender were categorized according to gender expression using the Socially Assigned Gender Expression scale (SAGE)^36^, a two-item, self-report measure based on (1) biological sex and (2) perceived gender expression (7-point scale ranging from very feminine—very masculine), which has been used in previous studies on nonconforming gender expression among adolescents^37^. Four groups were created: conforming gender expression (female youth who reported being somewhat, mostly or very feminine; male youth who reported being somewhat, mostly or very masculine), androgynous (youth who reported being equally feminine and masculine), moderately nonconforming gender expression (female youth who reported being somewhat masculine; male youth who reported being somewhat feminine), and highly nonconforming gender expression (female youth who reported being mostly or very masculine; male youth who reported being mostly or very feminine).

### Mental health measures

Past-month suicidal ideation was assessed via one item: “During the past 30 days, have you thought seriously about killing yourself?” Response options were: no; yes, but rarely; yes, some of the time; and yes, almost all of the time. Responses were dichotomized into no/yes, with affirmative responses to any of the yes response options categorized as yes.

Past-year suicide attempt was assessed by asking, “During the past 12 months, have you attempted to kill yourself?” Responses were no/yes.

Nonsuicidal self-injury was assessed through the question, “During the past 12 months, how many times did you do something to hurt yourself on purpose, without wanting to die, such as cutting or burning yourself?” Responses (0 times, 1 to 2 times, or 3 or more times) were dichotomized to never/ever.

A single-item assessed past-year depression: “During the past 12 months, did you ever feel so sad or hopeless almost every day for at least two weeks in a row that you stopped doing some usual activities?” Response options were no/yes.

Past-month anxiety was assessed through the Generalized Anxiety Disorder-2 (GAD-2)^38^, with higher scores reflecting more probable anxiety disorder. The GAD-2 is an empirically-validated measure which has shown acceptable reliability (α = 0.77) in prior research with youth participants^39^. In the current study, the psychometric properties of the GAD-2 were good (α = 0.86). Consistent with developer recommendations, participants who scored ≥ 3 (clinical cutoff) were classified as meeting anxiety screening criteria.

### Victimization measures

Past-month peer victimization was measured using the four-item victimization subscale from the University of Illinois Bully Scale, which has demonstrated high reliability in prior research (α = 0.88; example item, “Other students made fun of me.”)^40^. In the current sample, Cronbach’s alpha reliability coefficient was 0.86. Responses ranged from 1 = never to 4 = 5 or more times. Given extreme skew and kurtosis in the distribution of mean scores, responses were dichotomized to indicate none/any victimization.

Bias-based harassment was assessed with: “In the past 12 months, how often have you been bullied, threatened or harassed by others thinking you’re gay, lesbian, bisexual or transgender?” Responses were dichotomized to never/ever.

### Protective factors measures

Students’ perceived school-connectedness was measured using four items from the Psychological Sense of School Membership scale^41^, a four-item version found to be reliable (α = 0.79–0.85 across groups) in prior DCYA research^42^. In the current study, Cronbach’s alpha reliability coefficients ranged from 0.83–0.88 across transgender and nonconforming gender expression groups. Youth were asked, “How strongly do you agree or disagree with each statement about your school?” An example item was, “I feel like I belong at this school.” Response options were on a four-point Likert-type response scale ranging from 1 = strongly agree to 4 = strongly disagree. Responses were reverse coded, with higher scores indicating greater perceived school-connectedness. Mean scores were entered in analyses.

Perceived family support/monitoring was measured using a DCYA-specific seven-item scale, with items assessing monitoring (e.g., “My parents know where I am when I go out”) and support (e.g., “My parents talk with me about things that bother me”). The scale has good internal consistency (α = 0.80)^43^, and alpha reliability coefficients ranged from 0.82–0.88 across transgender and nonconforming gender expression groups in the present study. Response options were on a four-point Likert-type response scale ranging from 1 = always to 4 = never. Responses were reverse coded, with higher scores indicating greater perceived family support/monitoring. Mean scores were entered in analyses.

### Analytic strategy

Analyses were conducted in R versions 4.1.0 and 4.2.2^44^, and supplemental analyses were conducted in Stata 16^45^. Consistent with recommended screening approaches for assessing adolescent health disparities^46,47^, 91 youth were identified as potential “mischievous respondents” and removed from the sample. We also removed three respondents in the juvenile detention facility from the sample given that victimization experiences, mental health, and the putative protective factors are theorized to differ for students involved in the juvenile justice system. Following exclusion of these cases, there was a small amount of missingness on most sociodemographic variables (<2%) and all mental health variables (<5% for each item). Missingness on key variables was <10% for each item. In this study, a complete case analysis was used for all variables, including sociodemographic, exposure, outcome, and moderator variables. This approach is consistent with other studies using DCYA data. To provide a comprehensive consideration of findings, the Supplementary Methods and Supplementary Tables 1, 2, and 3 describe missingness in the data, and Supplementary Tables 4–7 present the results using multiple imputation (MI) procedures. Differences between complete case and MI analyses may be attributed to systematic missingness and biased imputations and therefore those results should be interpreted with caution. Supplementary Fig. 1 describes participant selection in this secondary data analysis. The total unweighted analytic sample for this study was 11,264 respondents.

First, the weighted proportions of mental health and victimization exposures were calculated for transgender youth and cisgender youth by gender expression groups. A series of chi-squared (χ^2^) tests examined omnibus differences between gender modality and gender expression groups for each binary mental health and victimization variable. Post hoc pairwise comparisons using the Bonferroni correction were conducted to formally test for specific proportional differences between groups for each binary mental health and victimization variable. Second, we estimated multivariable logistic regressions to examine if gender modality and gender expression were associated with each victimization and mental health variable, adjusting for potential confounders (age, biological sex, race/ethnicity). Cisgender youth with conforming gender expression were the reference group. Third, we stratified the sample and examined the effect of each victimization exposure variable on the odds of each mental health indicator, adjusting for the same covariates, among transgender youth. We ran parallel analyses for cisgender youth with nonconforming gender expression (inclusive of androgynous, moderately, and highly nonconforming youth). Fourth, moderators were examined with stratified multivariable logistic regressions, examining if each moderator (i.e., school-connectedness, family support/monitoring) buffered the impact of peer victimization – and separately, bias-based harassment – on mental health, adjusting for the same covariates. Parallel analyses were conducted for cisgender youth with nonconforming gender expression. All models used DCYA-provided survey weights, and a sandwich estimator approach to calculate robust standard errors was used to account for student clustering within schools. For null hypothesis testing, chi-squared tests were one-tailed; all other tests were two-tailed and a *p*-value < .05 was used to indicate statistical significance.

### Reporting summary

Further information on research design is available in the Nature Research Reporting Summary linked to this article.

## Results

### Sociodemographic characteristics

Analyzing the 2015 DCYA, a cross-sectional survey of students in Wisconsin’s second largest county, we found that 107 students (0.97%) reported identifying as transgender and 11,157 students (99.03%) were classified as cisgender in the final analytic sample. Further, 4.5% of the sample indicated they did not know what transgender means. Among cisgender youth, percentages for gender expression groups were: 87.1% gender conforming, 8.9% androgynous, 2.4% moderately nonconforming gender expression, and 1.7% highly nonconforming gender expression. In the analytic sample, mean age was 15.9 years (SD = 1.2); 51.7% reported a female sex, 72.6% identified as White, non-Hispanic, and 90.1% of students reported being straight/heterosexual. Table 1 provides additional sociodemographic results.

**Table 1:** Sociodemographic characteristics of the analytic sample.

Variable	*n* (%) (unweighted *N* = 11,264)
Age, mean (SD), y	15.9 (1.2)
δ14	1473 (12.7)
15	3120 (28.8)
16	2875 (25.9)
17	2585 (22.2)
ε18	1211 (10.4)
*Biological sex*	
Male	5494 (48.3)
Female	5770 (51.7)
*Race/ethnicity*	
White	8930 (72.6)
Black or African American	438 (6.9)
Hispanic or Latino	524 (7.4)
Asian, including Hmong	506 (5.6)
Multi-racial	664 (6.0)
Another race/ethnicity	202 (1.6)
*Sexual orientation*	
Straight or heterosexual	10243 (90.1)
Gay or lesbian	162 (1.5)
Bisexual	446 (4.4)
Questioning	238 (2.3)
Another sexual orientation	175 (1.8)
*Gender modality*	
Cisgender	11157 (99.03)
Transgender	107 (0.97)
*Gender expression groups*	
Conforming	9810 (87.1)
Androgynous	948 (8.9)
Moderately nonconforming	242 (2.4)
Highly nonconforming	157 (1.7)

Data Source: The Dane County Youth Assessment, 2015.

Percentage of respondents are weighted to be representative of the student population, whereas *n* is unweighted counts; percentages may not sum to 100 due to rounding. Participants who responded “No” to the gender modality item were classified by researchers as “cisgender.” The gender expression groups were researcher-classified using two items: biological sex and perceived gender expression. The classification excludes youth who responded “Yes” on the gender modality item.

### Victimization and mental health prevalence and disparities

Table 2 provides bivariate results; global bivariate statistical tests indicated significant group differences across all victimization and mental health indicators. Key findings from post hoc Bonferroni analyses indicated that a significantly greater proportion of transgender youth reported peer victimization than cisgender youth with conforming gender expression and highly gender nonconforming expression. A similar result was found for cisgender youth with moderately gender nonconforming expression compared to the same two groups; notably, over half of cisgender youth with moderately gender nonconforming expression reported peer victimization. For bias-based harassment, all groups reported significantly higher percentages of bias-based harassment than cisgender youth with gender conforming expression. In particular, nearly half of transgender youth and over one-third of cisgender youth with moderately gender nonconforming expression reported bias-based harassment, with both groups differing significantly from cisgender youth with gender conforming, androgynous, and highly nonconforming gender expressions.

**Table 2:** Victimization and mental health by gender expression groups and gender modality.

	Conforming		Androgynous		Moderately Non-conforming		Highly Non-conforming		Transgender		Global bivariate statistical tests	
	*n*	%	*n*	%	*n*	%	*n*	%	*n*	%	χ^2^ (df)	*p*-value
*Victimization*												
Peer victimization	4553	36.1^b^	587	45.8^a^	185	52.9^a^	78	32.8^b^	69	49.1^a^	93.90 (4)	**<0.001**
Bias-based harassment	801	6.4^b^	227	17.7^a,d^	130	37.3^a,c^	44	18.4^a,d^	68	48.4^a,c^	890.66 (4)	**<0.001**
*Mental health*												
Anxiety	4581	36.4^b^	637	49.8^a,c^	197	56.2^a,c^	83	34.8^d^	82	58.0^a,c^	163.72 (4)	**<0.001**
Depression	2479	19.7^b^	430	33.6^a,c,f^	124	35.6^a,c,f^	41	17.3^d,f^	76	54.0^a,e^	268.40 (4)	**<0.001**
Nonsuicidal self-injury	1627	12.9^b^	298	23.3^a,d^	105	30.1^a,c^	44	18.4^d^	55	39.2^a,c^	247.13 (4)	**<0.001**
Suicidal ideation	2014	16.0^b^	382	29.8^a,d^	131	37.5^a,c,d^	57	24.0^a,d^	85	60.2^a,c^	418.20 (4)	**<0.001**
Suicide attempt	461	3.7^b^	124	9.7^a,d^	40	11.5^d^	37	15.7^a^	31	21.9^a,c^	293.07 (4)	**<0.001**

Data Source: The Dane County Youth Assessment, 2015.

The gender expression groups were researcher-classified using two items: biological sex and perceived gender expression. This classification excludes youth who responded “yes” on the gender modality item. Gender modality consists of youth who reported “yes” on the gender modality item (i.e., transgender). Weighted percentages and weighted counts are representative of student population. Significance (*p* < 0.05) is noted in bold for global bivariate statistical tests (series of chi-squared tests). Significance for post hoc pairwise comparisons (Bonferroni tests) are denoted by superscript pairs (a,b), (c,d), and (e,f). Within the superscript pairs, “a” denotes a higher value than “b”, “c” denotes a higher value than “d”, and “e” denotes a higher value than “f”.

In regards to mental health, key findings from post hoc Bonferroni tests included that transgender youth reported significantly higher prevalence of depression (54%) and suicidal ideation (60.2%) relative to cisgender youth across all gender expression groups. Trangender youth also reported a significantly higher prevalence of suicide attempts (21.9%) than cisgender youth with gender conforming, androgynous, and moderately noncomforming gender expressions. A significantly higher proportion (58%) of transgender youth reported anxiety than cisgender youth with gender conforming and highly gender nonconforming expressions. Further, cisgender youth with moderately nonconforming gender expression were more likely to report anxiety, depression, nonsuicidal self-injury, and suicidal ideation than cisgender youth with conforming gender expression and with highly gender nonconforming expression. They also differed significantly from androgynous-classified youth in regards to nonsuicidal self-injury and suicidal ideation. Supplementary Table 5 presents bivariate results using MI procedures. Weighted proportions of each mental health and victimization indicator largely paralleled findings of the complete case analysis. Similarly, omnibus tests documented significant group differences for each binary mental health and victimization variable.

Table 3 provides multivariable results. Key findings included that transgender youth reported greater odds of all victimization and mental health distress indicators relative to cisgender youth with gender conforming expression. Transgender youth reported the highest odds of bias-based harassment (AOR = 14.57 [14.12, 15.01]), depression (AOR = 4.73 [4.39, 5.07]), nonsuicidal self-injury (AOR = 4.44 [3.87, 5.02]), suicidal ideation (AOR = 7.87 [7.53, 8.20]), and suicide attempt (AOR = 7.07 [6.51, 7.63]). Androgynous youth and youth with moderately nonconforming gender expression had elevated odds of peer victimization and bias-based harassment compared to cisgender youth with gender conforming expression. Relative to the reference group, youth with highly gender nonconforming gender expression had higher odds of bias-based harassment (AOR: 3.32 [2.86, 3.79]), but not peer victimization (AOR: 0.91 [0.22, 1.61]). Androgynous youth, and youth with moderately and highly gender nonconforming expression, had greater odds of each respective mental health indicator, with two exceptions; youth with highly gender nonconforming expression did not evidence significant differences on anxiety and depression.

**Table 3:** Logistic regressions predicting victimization and mental health by gender expression groups and gender modality.

	Androgynous		Moderately nonconforming		Highly nonconforming		Transgender	
	AOR	95% CI	AOR	95% CI	AOR	95% CI	AOR	95% CI
*Victimization*								
Peer victimization	1.56	1.43–1.70	2.10	1.82–2.39	0.91	0.22–1.61	1.77	1.27–2.27
Bias-based harassment	3.32	3.13–3.50	9.30	8.90–9.69	3.32	2.86–3.79	14.57	14.12–15.01
*Mental health*								
Anxiety	1.70	1.58–1.81	2.55	2.37–2.73	1.41	0.95–1.88	2.41	1.81–3.01
Depression	1.90	1.65–2.14	2.31	1.62–3.01	1.10	0.63–1.57	4.73	4.39–5.07
Nonsuicidal self-injury	1.99	1.78–2.19	3.33	3.07–3.58	2.45	1.97–2.94	4.44	3.87–5.02
Suicidal ideation	2.10	1.91–2.30	3.24	2.98–3.51	2.05	1.54–2.57	7.87	7.53–8.20
Suicide attempt	2.46	2.18–2.74	3.45	2.78–4.13	6.26	5.84–6.68	7.07	6.51–7.63

Data Source: The Dane County Youth Assessment, 2015.

The gender expression groups were researcher-classified using two items: biological sex and perceived gender expression. This classification excludes youth who responded “yes” on the gender modality item. Gender modality consists of youth who reported “yes” on the gender modality item (i.e., transgender). The AOR represents the odds of reporting the victimization or mental health outcome relative to cisgender youth with conforming gender expression (reference group) adjusting for age, biological sex, and race/ethnicity.

*AOR* adjusted odds ratio, *CI* confidence interval.

### Stratified analyses and protective factors

**Fig. 1:**
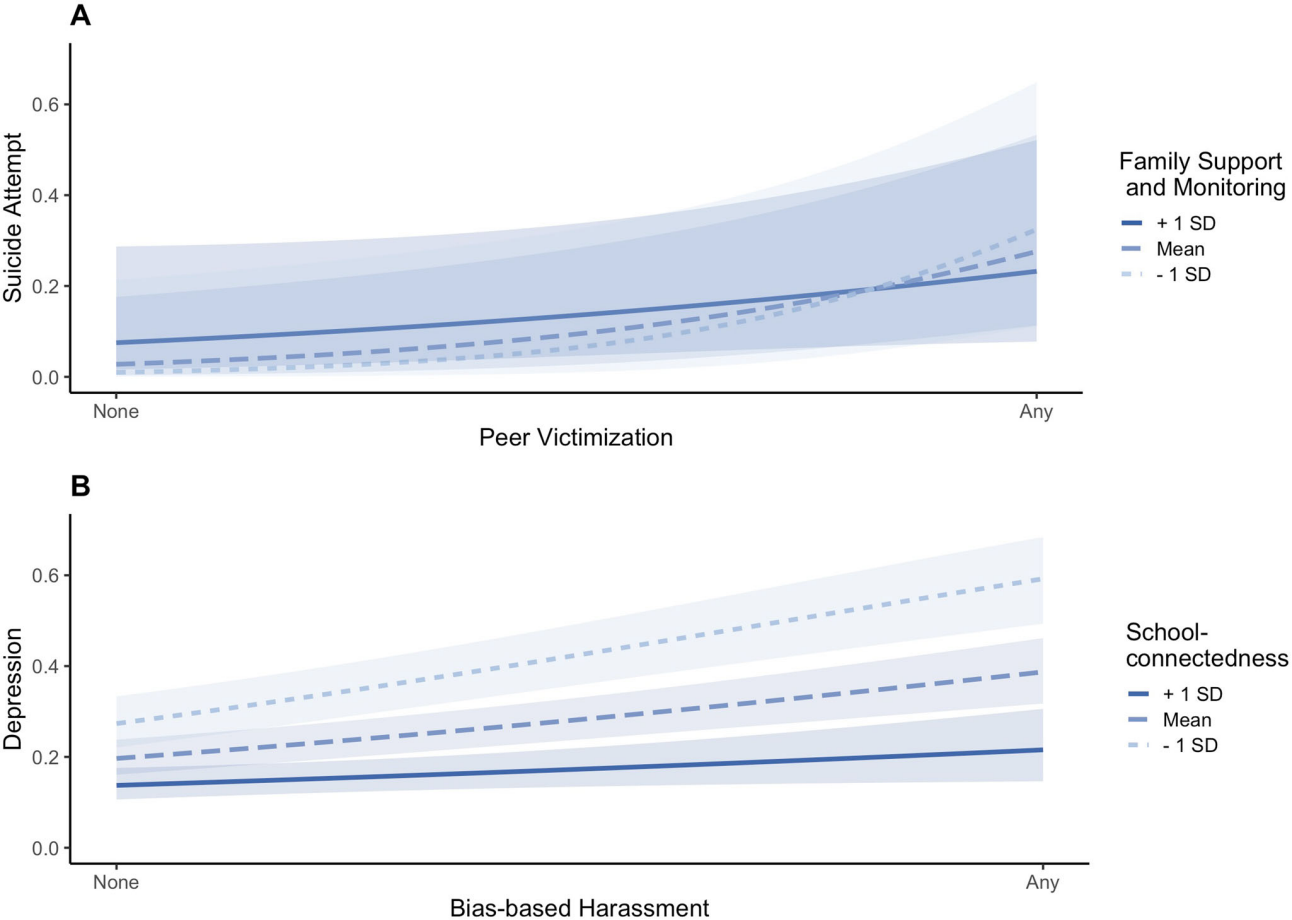
Predicted Probabilities of Mental Health Among Transgender Youth and Cisgender Youth with Nonconforming Gender Expression. **A** Stratified model of transgender youth, graphing the interaction effects of family support/monitoring (mean ± 1 standard deviation [SD]) and peer victimization for the predicted probability of suicide attempt. **B** Stratified model of cisgender youth with nonconforming gender expression, graphing the interaction effects of school-connectedness (mean ± 1 SD) and bias-based harassment for the predicted probability of depression. Data are weighted to be representative of the student population. Models are adjusted for age, biological sex, and race/ethnicity. Blue shaded ribbons represent 95% Confidence Intervals (CIs).

Stratified analyses among transgender youth revealed that peer victimization and bias-based harassment were related to all mental health indicators (Table 4). Tested protective factors (i.e., school-connectedness, family support/monitoring) did not moderate most associations, except for the interaction of peer victimization and family support/monitoring in predicting suicide attempts (Fig. 1A). Among transgender youth who reported peer victimization, those who reported lower levels of family support/monitoring (1 SD below the mean) were more likely to have reported a past-year suicide attempt than transgender youth reporting higher levels of family support/monitoring (1 SD above the mean).

**Table 4:** Logistic regressions predicting mental health among transgender youth reporting victimization and separately among cisgender youth with nonconforming gender expression reporting victimization.

	Anxiety		Depression		NSSI		SI		SA	
	AOR (95% CI)	z-value	AOR (95% CI)	z-value	AOR (95% CI)	z-value	AOR(95% CI)	z-value	AOR (95% CI)	z-value
*Transgender*										
Peer victimization	3.85 (2.82–4.88)		2.49 (1.53–3.44)		6.37 (5.42–7.31)		2.92 (2.12–3.71)		9.10 (7.63–10.57)	
x SC		−1.61		−1.74		0.74		−0.91		−0.28
x FSM		−0.40		0.39		−1.77		0.32		**−2.45**
BB harassment	3.99 (2.85–5.14)		2.37 (1.45–3.29)		5.04 (4.00–6.08)		2.72 (1.74–3.70)		9.68 (8.46–10.90)	
x SC		−1.31		0.26		1.94		−0.52		0.75
x FSM		−0.75		−1.89		−0.45		0.40		−0.27
*Nonconforming gender expression*										
Peer victimization	2.56 (2.35–2.76)		3.56 (3.37–3.76)		2.82 (2.29–3.35)		2.88 (2.63–3.14)		1.96 (1.66–2.25)	
x SC		−0.09		−0.03		−0.75		0.68		1.09
x FSM		−1.43		0.21		0.25		−0.35		−0.37
BB harassment	1.79 (1.32–2.26)		3.16 (2.82–3.50)		3.56 (3.18–3.93)		3.18 (2.74–3.62)		2.43 (2.03–2.84)	
x SC		−1.68		**−2.16**		1.04		−0.27		−1.18
x FSM		−0.47		0.20		0.38		0.37		0.62

Data Source: The Dane County Youth Assessment, 2015.

The AOR represents the odds of reporting the mental health outcome relative to the respective reference group who have not experienced the victimization indicator, adjusted for age, biological sex, and race/ethnicity. Significance (*p* < 0.05) noted in bold next to the reported z-value for interaction models (multiple logistic regression). Stratified analyses among cisgender youth with nonconforming gender expression include androgynous, moderately nonconforming, and highly nonconforming youth.

*AOR* adjusted odds ratio, *BB* harassment, bias-based harassment, *CI* confidence Interval, *FSM* family support/monitoring, *NSSI* nonsuicidal self-injury, *SC* school-connectedness, *SI* suicidal ideation, *SA* suicide attempt.

Parallel analyses among cisgender youth with nonconforming gender expression revealed that peer victimization and bias-based harassment were both significantly associated with all five psychological distress indicators (Table 4). The interaction of bias-based harassment and school-connectedness was significant in predicting past-year depression (Fig. 1B). Among adolescents with nonconforming gender expression who reported bias-based harassment, those who reported low school-connectedness (1 SD below the mean) were more likely to have reported depression than adolescents who experienced high school-connectedness (1 SD above the mean).

## Discussion

This investigation found that transgender youth and cisgender youth with nonconforming gender expression had increased odds of experiencing bias-based harassment, peer victimization, and severe mental health problems compared to their cisgender counterparts with conforming gender expression. The consistency of associations across domains underscores the high degree of distress and vulnerability in these subpopulations and the need for supports at multiple social-ecological levels.

Several novel contributions emerged. In studies of adolescents, information on gender modality or expression is infrequently gathered, leaving gaps in our understanding of gender diverse youth experiences. In research that assesses gender modality or perceived gender expression, few studies have disentangled the unique experiences of gender diverse youth subgroups or have combined sexual and gender diverse adolescents into a monolithic group. Our findings add to the literature through their nuanced consideration of the experiences of transgender and cisgender youth with nonconforming gender expression. Results augment past literature finding high prevalence and disparities in suicidal ideation, attempts, nonsuicidal self-injury, and victimization among transgender relative to cisgender youth^3–5,16,17^.

This study is one of the first to assess psychological functioning and victimization exposures among cisgender youth with nonconforming gender expression; findings suggest that level of gender nonconformity is a critical factor associated with wellbeing. Notably, androgynous youth and youth with moderately to highly gender nonconforming expression tended to report more victimization and psychological distress than cisgender gender conforming peers. Notably, moderately gender nonconforming youth reported more distress in some domains than highly gender nonconforming youth, a finding that is consistent with some previous research^37^. It might be that moderately gender nonconforming adolescents are in a process of gender exploration to a greater degree than highly gender nonconforming adolescents, who might be more likely to have increased confidence in their identities. In turn, such exploration could potentially heighten risk for distress and being targeted by harassment. Moderately gender nonconforming adolescents might therefore benefit from additional supports (e.g., Gender-Sexuality Alliances [GSAs], support from educators and staff) in the school context.

This study also bolsters the limited research on protective factors, with two moderator effects emerging. Among cisgender youth with nonconforming gender expression who experienced bias-based harassment, school-connectedness appears to protect against depression. Among transgender youth, family support/monitoring buffered the impact of peer victimization on suicide attempts. Despite other interactions being non-significant, school-connectedness and family support/monitoring each related to lower odds of mental health problems, but did not function differently for transgender youth and youth with nonconforming gender expression. Although interpreting non-significant associations is difficult, findings raise questions including whether the most relevant sources of adolescent supports were examined, if the quality of the family support/monitoring measure was poor, or if transgender youth and youth with nonconforming gender expression in the sample did not experience strong family support/monitoring, thus leaving them vulnerable to victimization and concomitant threats to wellbeing. It may also be that the psychological impact of victimization on gender diverse youth is of such severity that theorized protective factors cannot ameliorate the effects of specific victimization types (i.e., bias-based bullying), as similar research has found^48^.

The study is not without limitations. The sample was limited to one U.S. county, limiting the generalizability of findings. Further, the sample had limited racial and ethnic diversity, and as such findings do not capture the experiences of gender diverse youth who might experience victimization and marginalization due to both their gender identity or expression and their race/ethnicity. Assessments were self-report measures and a secondary data analysis was conducted, which precluded our ability to conduct more thorough psychiatric assessments with participants. Notably, the prevalence of mental health indicators, such as depression, was similar to prevalence estimates emerging from studies using either a physician-endorsed diagnosis or an empirically validated screening measure^49,50^. However, more reliable mental health data may have been collected using multi-item scales or clinical diagnostic interviews. The cross-sectional study design precluded our ability to determine temporality of the exposure and outcome variables. Longitudinal research would help elucidate the temporal ordering of victimization experiences and mental health. While we used a validated peer victimization scale, knowledge of which aspects of peer victimization were most salient is a potential limitation. Additionally, while school-connectedness and family support/monitoring were tested as protective factors, future DCYA surveys might include measures such as parental acceptance and support for gender identity. The sample size of transgender adolescents was small, precluding subgroup analyses in this group, and power was low in interaction models. Importantly, there were wide confidence intervals, and hence, limited statistical precision of estimates in the stratified models with transgender youth. Further, given the dataset was from 2015, findings do not capture the impact of the more recent intensified anti-transgender political climate across the United States on youth wellbeing. Lastly, research has demonstrated the methodological superiority of a two-step gender identity measure^51^. While we were limited by the survey’s single-item question asking if a respondent identified as transgender (thus precluding nonbinary and diverse gender identities), it is nonetheless valuable that this study included both gender modality and perceived gender expression.

Our findings, in tandem with a growing body of research on the mental health and victimization experiences of gender diverse young people, highlight the importance of bolstering services, supports, and structural factors across multiple levels of the social ecology. Further, our results point to the importance of supporting an often overlooked group of youth – cisgender gender nonconforming adolescents. At the societal-level, ensuring federal and state-level policies (e.g., enumerated anti-bullying laws) provide protections for transgender and gender diverse young people are sorely needed. The Movement Advancement Project (https://www.lgbtmap.org/equality-maps/safe_school_laws) and GLSEN (https://maps.glsen.org/enumerated-anti-bullying-and-harassment-policies) both provide timely and accurate monitoring of state anti-bullying laws that include protections for sexual and gender diverse youth^52,53^. In addition to advocacy efforts for anti-bullying policies, researchers should consider writing policy briefs and partnering with local, state, and national policymakers to ensure that all research findings on gender diverse youth guide the policy conversations that will impact the lives of youth most affected by such policies.

Schools and community-based organizations are optimally positioned to enhance positive outcomes among gender diverse youth. Universal programming focused on educating all youth about gender identity development, the diversity of gender identities and expressions, and use of inclusive language is recommended. Promoting inclusive curricula, including comprehensive sexual education that reflects the identities and experiences of gender diverse youth, is also an important practice^54^. Tailoring evidence-based prevention and intervention programs to meet the unique needs of gender diverse youth is warranted, particularly programming focused on ameliorating peer victimization and bullying given the high prevalence documented here. Given the importance of school-connectedness as an established protective factor among gender diverse youth, schools should encourage and empower educators to attend to both academic and social-emotional functioning of students, foster discussions on gender diversity and acceptance, and invite students as key stakeholders in classroom and school-wide efforts to enhance a sense of belonging and inclusion. Professional development for educators and staff should focus on disseminating knowledge on supporting gender-inclusive strategies in the classroom and creating a community of care^55^. Research has also shown that GSAs in schools are associated with improved well-being among gender diverse youth^31^. Providing funding for active GSAs and supporting students in initiating new clubs or peer groups where they have previously not existed is recommended as a key step in augmenting the health and well-being of gender diverse youth.

Similarly, mental health practitioners, including school-based clinicians, should provide tailored, evidence-based interventions to mitigate the mental health and victimization disparities documented in this study. Given that some of our findings point to heightened risk for moderately gender nonconforming cisgender youth, clinicians should be aware of some of the unique experiences such youth might face. To support optimal care, practitioners should receive culturally competent training in the gender-affirmative care model^56^, and it is essential for healthcare providers to receive training focused on gender diversity^57^. Implications for pediatricians seeking to bolster gender diverse adolescents’ wellbeing and safety include routine screening for mental health and victimization at well-being visits. Collaborative care across medical specialties and/or referral to specialists for gender diverse youth may also be an important avenue in affirming and supporting youth^56^.

Additionally, our findings on the protection conferred by family support and monitoring dovetail with the broader literature^58^ and suggest that strategies which bolster family support are recommended. This is in line with recommendations from The Society for Adolescent Mental Health and Medicine, which include the promotion of family connections, as well as acceptance, for GD youth^59^. Researchers might partner with schools and community-based organizations to offer seminars or workshops (e.g., family forums) to educate families and the general public on the relationship between strong family support and well-being among gender diverse youth. Further, it is essential for researchers to disseminate such findings in creative ways, ranging from snapshot reports that could be shared with schools, community agencies, and pediatricians’ offices, to easily digestible key points disseminated through social media. Lastly, the considerable strength and resilience of transgender youth, as well as cisgender youth with nonconforming gender expressions, should continue to be promoted.

In sum, this study documented high prevalence and marked disparities of psychiatric distress and victimization among gender diverse youth. School-connectedness buffered the impact of bias-based harassment on depression among youth with gender nonconforming expression, and family support/monitoring emerged as a protective factor among transgender youth. Future research should attend to the intersection of multiple marginalized identities and their relation to mental health. Longitudinal studies are needed to elucidate mechanisms and understand how violence victimization in adolescence impacts gender diverse wellbeing across the lifespan. Further, research examining protective factors across multiple social-ecological levels would enhance our ability to address disparities and bolster targeted prevention and intervention efforts among gender diverse adolescents.

### Supplementary information

Supplementary Information

Reporting Summary

STROBE Statement

## Data Availability

The data that support the findings of this study are available from the Dane County Youth Commission, but restrictions apply to the availability of these data and thus are not publicly available. Researchers interested in accessing these data can do so with permission of the Dane County Youth Commission.

## Code availability

Open-source code for this study is available at https://github.com/katharineparodi/dcya-2015-npj. Analyses were conducted using R versions 4.1.0 and 4.2.2.
