# Supplementary material for: Identifying protective factors for gender diverse adolescents’ mental health
Source: Npj Ment Health Res. 2023 Jul 4;2:10. doi: 10.1038/s44184-023-00029-8 (PMC10955934; doi:10.1038/s44184-023-00029-8)
Supplement: Supplementary file 1 — Supplementary Information [file 44184_2023_29_MOESM1_ESM.pdf]

Supplementary Information

Supplementary Methods

Supplementary Table 1 shows the amount (%) of missing data for each variable of interest. The variables most affected by missing data were peer victimization (9.7%), gender conformity level (8.1%), school connectedness (7.6%) and bias-based harassment (7.6%). Of the 14 variables used in the analysis, 10 had missing data for less than 4.2% of the sample.

Following the guidelines of Sterne et al.[1], we attempted to use multiple imputation was to account for these missing data under a missing at random (MAR) assumption. However, the evidence to support the MAR assumption was mixed, as evidenced by the many differences between complete cases and cases with missing data (Supplementary Table 2). Examination of missing value patterns revealed that 83% of the sample had complete data on all variables, 2% were missing data on four of the five mental health outcomes (depression, non-suicidal self-injury, suicidal ideation and suicide attempt), and 2% were missing data on sexual orientation (Supplementary Table 3). Another 231 patterns of missing data were each found in less than 1% of the sample.

Imputation of 20 datasets was performed using chained equations in Stata 16 (command: *mi impute chained*). This approach fills in missing values iteratively by using a sequence of univariate imputation methods with fully conditional specification of prediction equations.[2] The categorial variables predicted using a logit link were: race/ethnicity, sexual orientation, gender conformity level (including transgender), peer victimization, bias-based harassment, anxiety, depression, non-suicidal self-injury, suicidal ideation and suicide attempt. Age was entered as an ordinal variable and predicted using an ordered logit link. The continuous variables that were imputed using predictive mean matching were family support/monitoring and school connectedness. Weighted logistic regression analysis within Stata's MI toolkit (command: *mi estimate*) was then used to combine estimates across the 20 datasets following Rubin's rules.[3] Standard errors were adjusted to account for within-school clustering using Stata's *vce(cluster school)* option.

With imputed data, the distributions found in key variables were similar to those in the complete case analysis (Table 1 and Supplementary Table 4), as were prevalence estimates of mental health problems across gender expression groups (Table 2 and Supplementary Table 5). However, we observed wider confidence intervals around odds ratios of victimization and mental health (Supplementary Table 6) which may explain why interactions of victimization and two support variables were consistently non-significant (Supplementary Table 7). We cannot rule out the possibility of biased imputations given the differences between imputed and non-imputed data, and therefore urge caution when interpreting these results.

Supplementary Table 1. Missing Data on Key Variables (n=13,811).

Variable	Missing	Imputed cases (n)
Age	0.2%	31
Sex	0.8%	116
Race/ethnicity	2.5%	345
Sexual orientation	1.2%	161
Gender conformity level	8.1%	1123
School connectedness	7.6%	1053
Family support and monitoring	4.1%	569
Peer victimisation	9.7%	1339
Bias-based harassment	7.6%	1056
Anxiety	3.8%	520
Depression	4.2%	577
Non-suicidal self-injury	4.1%	568
Suicidal ideation	4.2%	579
Suicide attempt	4.1%	566

Supplementary Table 2. Unweighted Differences between Individuals with and without Missing Data on Key Variables.

Variable		Not missing (n=11,264)	Imputed cases (n=2547)	P-value
Age, y	mean	15.9	15.8	<0.001
≤14	%	12.7	15.3	0.017
15	%	28.8	30.1	0.014
16	%	25.9	25.6	0.720
17	%	22.2	18.9	<0.001
≥18	%	10.4	10.1	0.533
Biological sex				
Male	%	48.3	57.3	<0.001
Female	%	51.7	42.7	<0.001
Race/ethnicity				
White	%	72.6	62.9	<0.001
Black or African American	%	6.9	12.1	<0.001
Hispanic or Latino	%	7.4	9.7	<0.001
Asian, including Hmong	%	5.6	3.1	0.334
Multi-racial	%	6.0	9.1	<0.001
Another race/ethnicity	%	1.6	3.0	<0.001
Sexual orientation				
Straight or heterosexual	%	90.1	87.9	<0.001
Gay or lesbian	%	1.5	1.8	0.181
Bisexual	%	4.4	4.8	0.057
Questioning	%	2.3	2.7	0.057
Another sexual orientation	%	1.8	2.9	<0.000
Gender modality				
Cisgender	%	99.03	96.8	<0.001
Transgender	%	0.97	3.2	<0.001
Gender expression groups				
Conforming	%	87.1	75.4	<0.001
Androgynous	%	8.9	12.8	<0.001
Moderately nonconforming	%	2.4	2.9	0.078
Highly nonconforming	%	1.7	3.7	<0.001
Victimization				
Peer victimization	%	38.1	36.4	0.250
Bias-based harassment	%	8.72	12.4	<0.001
Protective factors				
School connectedness	Mean	3.1	3.0	<0.001
Family Support/Monitoring	Mean	3.4	3.2	<0.001
Mental Health				
Anxiety	%	37.5	40.0	0.042
Depression	%	20.3	25.2	<0.001
Nonsuicidal self-injury	%	13.9	16.7	0.002

Suicidal ideation	%	17.7	20.0	0.018
Suicide attempt	%	4.5	7.3	<0.001

Note. Differences were tested using t-tests for continuous variables and chi-squares for categorical variables.

Supplementary Table 3. Missing Value Patterns.

%	Pattern													
	Age	Sex	RE	SO	GCI	SC	FSM	PV	BBH	ANX	DEP	NSSI	SI	SA
83	1	1	1	1	1	1	1	1	1	1	1	1	1	1
2	1	1	1	1	1	1	1	1	1	1	0	0	0	0
2	1	1	1	0	1	1	1	1	1	1	1	1	1	1
1	1	1	1	1	0	0	0	0	0	0	0	0	0	0
1	1	1	1	1	1	1	1	0	1	1	1	1	1	1
1	1	1	1	1	1	1	1	1	1	1	1	1	0	1
1	1	1	1	1	1	1	1	1	1	1	0	1	1	1
<1	1	1	1	1	1	1	1	1	1	1	1	1	1	0
<1	1	1	1	1	0	0	0	1	0	0	0	0	0	0
<1	1	1	1	1	1	1	1	1	1	1	1	0	0	0
<1	1	1	1	1	1	1	1	1	1	1	1	0	1	1
<1	1	1	1	1	1	1	1	1	1	1	1	0	1	0
<1	1	1	1	1	0	1	1	1	1	1	1	1	1	1

Note: 1 indicates non-missing data and 0 indicates missing data. The first row shows non-missing data in 83% of the sample. RE: Race/ethnicity, SO: Sexual orientation, GFI: Gender conformity level, SC: School connectedness, FSM: Family support and monitoring, PV: Peer victimization, BBH: Bias-based harassment, ANX: Anxiety, DEP: Depression, NSSI: Non-suicidal self-injury, SI: Suicidal ideation, SA: Suicide attempt. Not shown are 227 more patterns, each found in less than 1% of the sample.

Supplementary Table 4. Sociodemographic Characteristics of the Analytic Sample with Multiple Imputation

Variable	n (%)<br>(unweighted N = 13,811)
Age, mean (SD), y	15.9 (1.2)
≤14	1898 (13.7)
15	3918 (28.4)
16	3494 (25.3)
17	3052 (22.1)
≥18	1449 (10.5)
Biological sex	
Male	6825 (49.4)
Female	6986 (50.6)
Race/ethnicity	
White	10711 (77.6)
Black or African American	720 (5.2)
Hispanic or Latino	773 (5.6)
Asian, including Hmong	487 (3.5)
Multi-racial	890 (6.4)
Another race/ethnicity	231 (1.7)
Sexual orientation	
Straight or heterosexual	12491 (90.4)
Gay or lesbian	202 (1.5)
Bisexual	554 (4.0)
Questioning	301 (2.2)
Another sexual orientation	263 (1.9)
Gender modality	
Cisgender	13629 (98.7)
Transgender	182 (1.3)
Gender expression groups	
Conforming	11831 (85.7)
Androgynous	1239 (9.0)
Moderately nonconforming	310 (2.2)
Highly nonconforming	249 (1.8)

Data Source: The Dane County Youth Assessment, 2015
Note. Percentage of respondents are weighted to be representative of the student population, whereas *n* is unweighted counts; percentages may not sum to 100 due to rounding. Participants who responded "No" to the gender modality item were classified by researchers as "cisgender." The gender expression groups were researcher-classified using two items: biological sex and perceived gender expression. The classification excludes youth who responded "Yes" on the gender modality item.

Supplementary Table 5. Victimization and Mental Health by Gender Expression Groups and Gender Modality with Multiple Imputation

	Conforming		Androgynous		Moderately Non-conforming		Highly Non-conforming		Transgender		Global bivariate statistical tests	
	n	%	n	%	n	%	n	%	n	%	χ^2 (df)	p-value
Victimization												
Peer victimization	4957	36.9	6180	44.7	7394	53.5	4748	34.4	6847	49.6	125.56 (4)	**<0.001**
Bias-based harassment	940	6.8	2470	17.9	4785	34.6	2743	19.9	6872	49.8	908.48 (4)	**<0.001**
Mental Health												
Anxiety	4309	36.4	617	49.8	169	54.6	90	36.2	114	62.6	230.15 (4)	**<0.001**
Depression	2390	20.2	431	34.8	104	33.8	55	22.0	94	51.3	358.06 (4)	**<0.001**
Nonsuicidal self-injury	1535	13.0	300	24.2	90	29.1	51	20.7	82	45.1	413.98 (4)	**<0.001**
Suicidal ideation	1914	16.2	372	30.0	116	37.6	73	29.2	105	57.5	523.53 (4)	**<0.001**
Suicide attempt	484	4.1	133	10.7	40	13.0	45	18.2	47	25.8	440.85 (4)	**<0.001**

Data Source: The Dane County Youth Assessment, 2015

Note. The gender expression groups were researcher-classified using two items: biological sex and perceived gender expression. This classification excludes youth who responded “yes” on the gender modality item. Gender modality consists of youth who reported “yes” on the gender modality item. Weighted percentages and weighted counts are representative of student population. Significance ($p < 0.05$) noted in bold for global bivariate statistical tests (series of chi-squared tests).

Supplementary Table 6. Logistic Regressions Predicting Victimization and Mental Health by Gender Expression Groups and Gender Modality with Multiple Imputation

	Androgynous		Moderately Nonconforming		Highly Nonconforming		Transgender	
	AOR	95% CI	AOR	95% CI	AOR	95% CI	AOR	95% CI
Victimization								
Peer victimization	1.47	1.26 – 1.70	2.03	1.51 – 2.72	1.02	0.61 – 1.70	1.73	1.13 – 2.64
Bias-based harassment	2.97	2.35 – 3.75	7.28	5.11 – 10.37	3.44	2.16 – 5.46	14.57	9.71 – 21.87
Mental Health								
Anxiety	1.68	1.45 – 1.94	2.44	1.94 – 3.05	1.56	1.08 – 2.25	3.18	1.88 – 5.38
Depression	1.98	1.58 – 2.48	2.06	1.20 – 3.52	1.42	0.96 – 2.11	4.20	3.00 – 5.90
Nonsuicidal self-injury	2.07	1.68 – 2.53	3.11	2.44 – 3.96	2.86	1.86 – 4.40	6.00	3.48 – 10.36
Suicidal ideation	2.09	1.70 – 2.58	3.09	2.35 – 4.07	2.64	1.84 – 3.80	7.26	5.17 – 10.21
Suicide attempt	2.36	1.79 – 3.10	3.15	1.54 – 6.45	5.82	3.94 – 8.59	7.41	4.48 – 12.23

Data Source: The Dane County Youth Assessment, 2015

AOR, Adjusted Odds Ratio; CI, Confidence Interval

Note. The gender expression groups were researcher-classified using two items: biological sex and perceived gender expression. This classification excludes youth who responded “yes” on the gender modality item. Gender modality consists of youth who reported “yes” on the gender modality item.The AOR represents the odds of reporting the victimization or mental health outcome relative to cisgender youth with conforming gender expression (reference group) adjusting for age, biological sex, and race/ethnicity.

Supplementary Table 7. Logistic Regressions Predicting Mental Health among Transgender Youth Reporting Victimization and Separately among Cisgender Youth with Nonconforming Gender Expression Reporting Victimization with Multiple Imputation

	Anxiety		Depression		NSSI		SI		SA	
	AOR (95% CI)	t-score	AOR (95% CI)	t-score	AOR (95% CI)	t-score	AOR (95% CI)	t-score	AOR (95% CI)	t-score
Transgender										
Peer victimization	3.67 (1.29 – 10.45)		3.03 (1.29 – 7.08)		4.53 (1.72 – 11.89)		3.52 (1.50 – 8.26)		4.84 (1.41 – 16.68)	
x SC		-1.12		-1.14		-0.15		-0.84		-0.94
x FSM		-0.20		0.10		-0.39		0.16		-0.89
BB harassment	4.10 (1.36 – 12.34)		2.91 (1.24 – 6.80)		4.30 (1.88 – 9.89)		3.21 (1.40 – 7.34)		5.66 (1.77 – 18.07)	
x SC		-1.13		0.19		0.91		-0.52		0.32
x FSM		-0.62		1.20		-0.74		0.32		-0.71
Nonconforming gender expression										
Peer victimization	2.47 (2.00 – 3.05)		3.12 (2.37 – 4.09)		2.49 (1.52 – 4.09)		2.63 (1.86 – 3.71)		1.90 (1.23 – 2.95)	
x SC		-0.90		-0.95		-1.33		-0.87		-0.20
x FSM		0.87		0.54		0.77		-0.06		-0.33
BB harassment	2.03 (1.39 – 2.98)		2.93 (2.21 – 3.88)		3.03 (2.12 – 4.34)		3.08 (2.21 – 4.30)		2.36 (1.45 – 3.82)	
x SC		-0.73		-1.29		-1.15		-0.15		-0.24
x FSM		-0.43		1.18		1.10		-0.01		-0.56

Data Source: The Dane County Youth Assessment, 2015

AOR, Adjusted Odds Ratio; BB harassment, Bias-based harassment; CI, Confidence Interval; FSM, Family Support/Monitoring; NSSI, Nonsuicidal Self-Injury; SC, School-Connectedness; SI, Suicidal Ideation; SA, Suicide Attempt

Note. The AOR represents the odds of reporting the mental health outcome relative to the respective reference group who have not experienced the victimization indicator, adjusted for age, biological sex, and race/ethnicity. Stratified analyses among cisgender youth with nonconforming gender expression include androgynous, moderately nonconforming, and highly nonconforming youth.

Supplementary References

1. Sterne JA, White IR, Carlin JB, et al. Multiple imputation for missing data in epidemiological and clinical research: potential and pitfalls. BMJ 2009;338:b2393.

2. White IR, Royston P, Wood AM. Multiple imputation using chained equations: Issues and guidance for practice. Stat Med 2011;30(4):377-99.

3. Rubin DB. Multiple imputation for nonresponse in surveys. New York, NY: Wiley 1987.

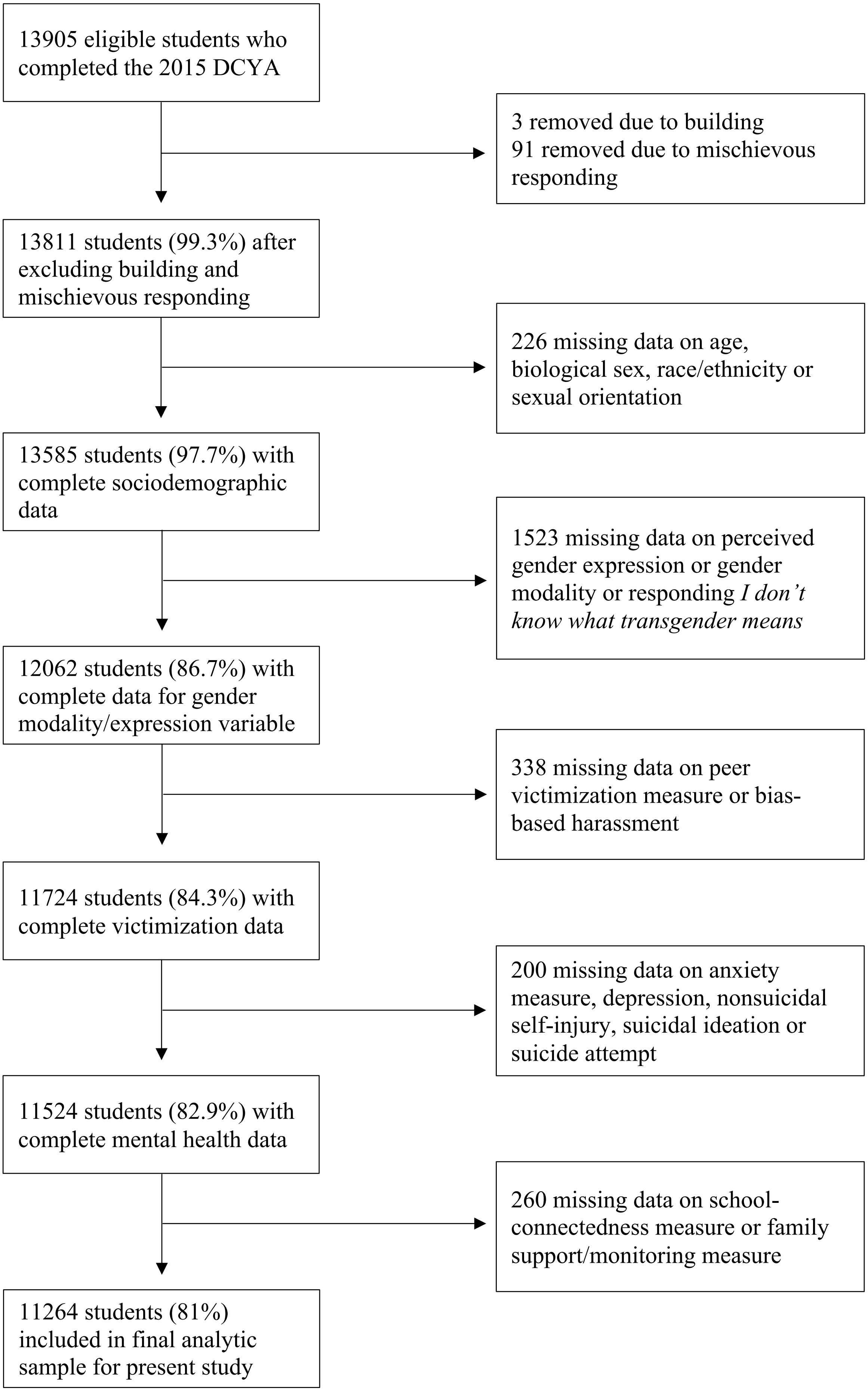

Supplementary Figure 1. Flow Diagram for Participant Selection. The flowchart shows the selection of study participants from the 2015 Dane County Youth Assessment which comprise the analytic sample.
